# Optimal Machining Strategy Selection in Ball-End Milling of Hardened Steels for Injection Molds

**DOI:** 10.3390/ma12060860

**Published:** 2019-03-14

**Authors:** Irene Buj-Corral, Jose-Antonio Ortiz-Marzo, Lluís Costa-Herrero, Joan Vivancos-Calvet, Carmelo Luis-Pérez

**Affiliations:** 1 Universitat Politècnica de Catalunya (UPC)-Escola Tècnica Superior d’Enginyeria Industrial de Barcelona (ETSEIB), 08034 Barcelona, Spain; irene.buj@upc.edu (I.B.-C.); jose.antonio.ortiz@upc.edu (J.-A.O.-M.); lluis.costa@upc.edu (L.C.-H.); joan.vivancos@upc.edu (J.V.-C.); 2 Universidad Pública de Navarra-Dpto. de Ingeniería, 31006 Navarra, Spain

**Keywords:** surface finish, high speed milling (HSM), roughness, modeling

## Abstract

In the present study, the groups of cutting conditions that minimize surface roughness and its variability are determined, in ball-end milling operations. Design of experiments is used to define experimental tests performed. Semi-cylindrical specimens are employed in order to study surfaces with different slopes. Roughness was measured at different slopes, corresponding to inclination angles of 15°, 45°, 75°, 90°, 105°, 135° and 165° for both climb and conventional milling. By means of regression analysis, second order models are obtained for average roughness *Ra* and total height of profile *Rt* for both climb and conventional milling. Considered variables were axial depth of cut *a_p_*, radial depth of cut *a_e_*, feed per tooth *f_z,_* cutting speed *v_c,_* and inclination angle *Ang*. The parameter *a_e_* was the most significant parameter for both *Ra* and *Rt* in regression models. Artificial neural networks (ANN) are used to obtain models for both *Ra* and *Rt* as a function of the same variables. ANN models provided high correlation values. Finally, the optimal machining strategy is selected from the experimental results of both average and standard deviation of roughness. As a general trend, climb milling is recommended in descendant trajectories and conventional milling is recommended in ascendant trajectories. This study will allow the selection of appropriate cutting conditions and machining strategies in the ball-end milling process.

## 1. Introduction

In order to increase productivity and reduce costs, it is important to choose appropriate cutting conditions in high speed milling (HSM) processes because they will influence surface roughness and the dimensional precision obtained. For example, the tool inclination angle significantly influences the surface roughness obtained. When the tool is perpendicular to the workpiece’s surface, cutting speed is zero at the tool tip [1,2]. This implies that the tool tends to crush the material instead of cutting it. 

In mathematical modeling of machining processes several methods can be used, such as statistical regression techniques, artificial neural network modeling techniques (ANN), and fuzzy set theory-based modeling [3]. Neural networks provide a relationship between input and output variables by means of mathematical functions, to which different weights are applied. A training algorithm is defined that consists of adjusting the weights of a network that minimize error between actual and desired outputs [4]. In recent times, neural networks have been used for modeling and predicting surface roughness in different machining operations. For example, Feng et al. modeled roughness parameters related to the Abbott–Firestone curve by means of ANN in honing operations [5] and in turning processes [6]. Özel et al. [7] and Sonar et al. [8] also employed ANN for modeling average roughness *Ra*, in turning processes. Moreover, simulations of machined surfaces have also been extensively investigated. Among many other studies, T. Gao et al. [9] developed a new method for the prediction of the machined surface topography in the milling process and Honeycutt and Schmitz [10] employed time domain simulation and experimental results for surface location error and surface roughness prediction. Vallejo and Morales-Menendez [11] used neural networks for modeling *Ra* in peripheral milling, with different input variables, such as feed per tooth, cutting tool diameter, radial depth of cut, and Brinell hardness. Zain et al. [12] modeled surface roughness with cutting speed, feed rate and radial rake angle as input variables in peripheral milling, and Quintana et al. [13] employed neural networks for studying average roughness in vertical milling. Regarding ball-end milling processes, Zhou et al. [14] used grey relational analysis (GRA) with neural network and particle swarm (PSO) algorithm to model 3D root mean square deviation of height value *Sq*, and compressive residual stresses, with tilt angle, cutting speed and feed as variables.

With regard to the modeling of milling processes by conventional regression models, several models have been developed, but most studies do not consider the variability which occurs as a consequence of the slope variations and which is developed in this study. Vivancos et al. [15] obtained mathematical models for arithmetic average roughness in ball-end milling operations by means of design of experiments, while Dhokia et al. [16] used design of experiments in ball-end milling to obtain models as a function of speed, feed and depth of cut. Oktem et al. [17] searched for minimum values in end milling taking into account cutting speed, feed rate, axial and radial depth of cut, and machining tolerance as input variables. In addition, they compared a response surface model with a neural network model [18]. It was observed that ANN lead to more accurate models than response surface methodology (RSM). Karkalos et al. [19] also compared regression models with ANN models in ball-end milling, with cutting speed, feed and depth of cut as variables and surface roughness as response. They found a higher correlation coefficient for ANN models than for RSM models. Vakondios et al. [20] obtained third order regression models for average maximum height of the profile *Rz*, as a function of axial depth of cut, radial depth of cut, feed rate and inclination angle, taking into account different manufacturing strategies. Wojciechowski and et al. [21] obtained a model for determining cutter displacements in ball-end milling. They took into account cutting conditions, surface inclination angle, run out, and the tool’s deflection. They found that both the cutter’s runout and surface inclination strongly influence cutter displacement. Wojciechowski and Mrozek optimized cutting forces and efficiency of the ball-end milling as a function of cutting speed and surface inclination angle [22]. Regarding Taguchi design of experiments, Pillai et al. [23] optimized machining time and surface roughness as a function of tool path strategic, spindle speed and feed rate in end milling with a single flute tool.

The main purpose of this study is to select an optimal machining strategy between climb and conventional milling in ball-end milling processes. For doing this, first mathematical models for roughness as a function of main process parameters were found. Unlike other works, in the present paper inclination angle of the surface to be machined is taken into account. Specifically, regression models and neural network models were obtained for parameters average roughness *Ra*, and total height of profile *Rt*. Finally, an optimal machining strategy was selected between climb and conventional milling for the different inclination angles considered. This will help molds and dies manufacturers to select appropriate strategies and cutting conditions in finish operations of surfaces with different inclination angles.

## 2. Experimental Procedure

### 2.1. Milling Tests

In the present study a factorial design of experiments was used for selecting experimental conditions in the ball-end milling process. The purpose of experimental tests is to analyze variability in the machining process of parts for injection molds, by means of several measurements performed on different areas of the machined workpieces with different inclinations. Two strategies were considered: climb milling and conventional milling.

**Figure 1:**
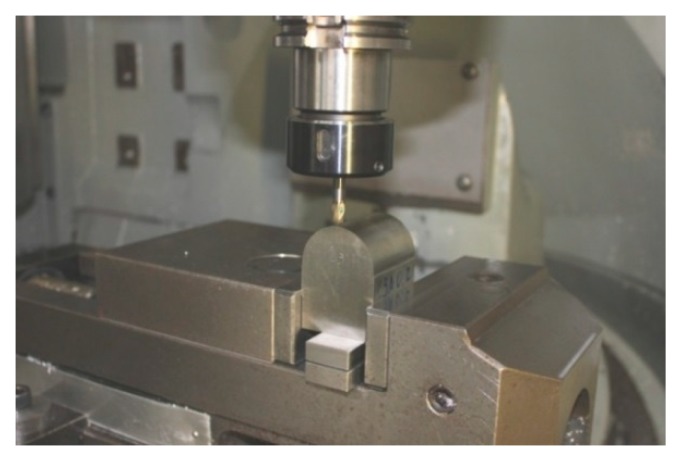
Manufactured part and high-speed machining center employed.

The workpieces were manufactured in an HSM center with vertical-spindle Deckel Maho DMU 50 Evolution (DMG Mori Seiki Co, Nakamura-ku, Nagoya, Japan) with Heidenhain control TNC 430 (Dr. Johannes Heidenhain GmbH, Traunreut, Germany), as shown in Figure 1, and tool holder MST Ref. DN40AD-CTH20-75. Tool details are presented in Table 1. 

Tools employed were new or had little wear (average flank wear VB < 0.1 mm), in order to avoid influence of wear on surface roughness. Only three axes (X-Y-Z) were used.

**Figure 2:**
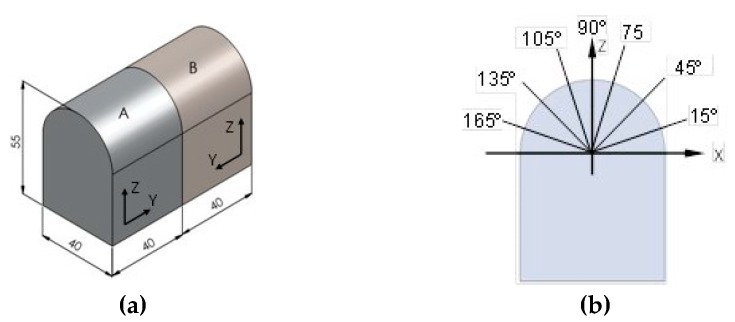
Schematic drawing of (**a**) machined workpiece (units in mm), (**b**) measured position angles.

Semi-cylindrical workpieces were machined in order to assess the effect of slope on surface roughness (Figure 2a). The material used for manufacturing the parts was a hot work tool steel W-Nr. 1.2344, hardened steel (50-54 HRC), with an approximate composition of 0.39% C, 1.10% Si, 0.40% Mn, 5.20% Cr, 1.40% Mo and 0.95% V.

A central composite design was chosen for modeling the behavior of both *Ra* and *Rt*, consisting of a two level factorial design with 4 factors (2^4^ = 16 experiments), with 4 central points. Since first-order models turned out to be inadequate for modeling both behavior of *Ra* and *Rt*, 8 star points were added, thus providing an orthogonal design with star points located at an axial distance of 1.60717. Selected factors were feed per tooth (*f_z_*), axial depth (*a_p_*), cutting speed (*v_c_*), and radial depth (*a_e_*). The study was developed for finish machining. Low and high levels for the different factors are shown in Table 2. 

For each experiment, roughness was measured at different angular positions corresponding to different inclination angles of the workpiece’s surface, as explained in Section 2.2 (Figure 2).

### 2.2. Roughness Measurement

**Figure 3:**
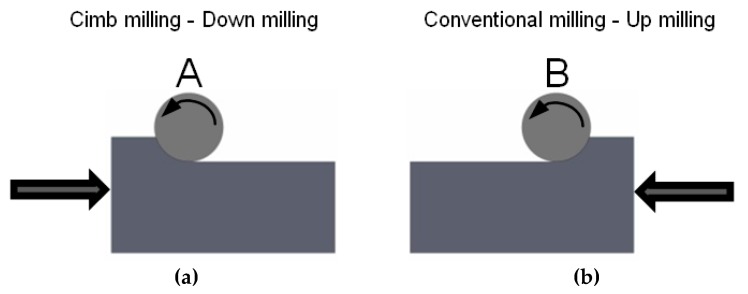
Schematic drawing of milling strategies. (**a**) climb (down) milling; (**b**) conventional (up) milling.

Roughness was measured along different generatrices of the semi-cylindrical part in Figure 2a, corresponding to different angular positions (15°, 45°, 75°, 90°, 105°, 135° and 165°) in Figure 2b. Moreover, influence of milling strategy, either climb (down) milling (Figure 3a) or conventional (up) milling (Figure 3b), on surface roughness was also analyzed (Figure 3).

**Figure 4:**
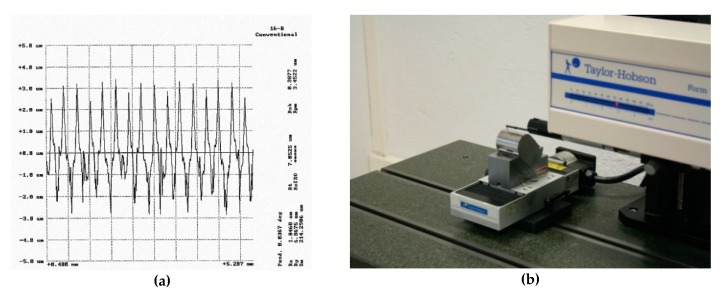
(**a**) Example of roughness profile; (**b**) Taylor-Hobson Form Taylsurf Series 2 profile roughness tester.

Roughness parameters *Ra* and *Rt* were measured using a Taylor-Hobson Form Taylsurf Series 2 profile roughness tester (as Figure 4b shows). An evaluation length of 4.8 mm (6 × 0.8 mm) was used, and a 2 μm radius stylus tip was used in conjunction with a 0.8 Gaussian cut-off filter and a bandwidth ratio of 320:1 to evaluate the *Ra* and *Rt* parameters. A stylus speed of 0.5 mm/s was used in conjunction with a 0.8 mN static stylus force and the stylus cone angle used was 90°.

Figure 4a shows an example of a roughness profile. A quite regular profile with higher peaks than valleys was observed, which corresponds to ball-end milling. Although a high number of roughness parameters were measured, as shown in Figure 4a, parameters *Ra* and *Rt* were selected in order to obtain results related to a high averaging parameter (*Ra*) and a low averaging parameter (*Rt*). In addition, both roughness parameters are commonly used in roughness characterization [13,24]. 

### 2.3. Photographs

A Leica S8AP0 binocular magnifier (Leica Camera AG, Wetzlar, Germany) was used to obtain photographs of the workpiece’s surface at 80× magnification.

## 3. Surface Roughness Results

In Table 3, as an example, roughness values of experiment 16 are compared, considering both A and B manufacturing strategies, respectively, at different angles which correspond to ascendant and descendant trajectories. Experiment 16 was chosen because it corresponds to high *a_p_*, *a_e_*, *f_z_*, and ***v_c_*** values (cutting conditions shown in Table 2), which lead to higher roughness values. In the images, changes of surface topography can be observed as a function of machining strategy (conventional or climb milling), position angle of the machined surface, and whether the tool displacement along *f_z_* trajectory is ascendant or descendant. According to the methodology explained in Section 2, different slopes of the machined semi-cylindrical workpieces were considered. For 15°, 45° and 75° in climb milling (Figure 3a), corresponding to 165°, 135° and 105° in conventional milling (Figure 3b), the tool displacement is ascendant. For 105°, 135° and 165° in climb milling, corresponding to 75°, 45° and 15° in conventional milling, the tool displacement is descendant.

In climb milling, roughness values remain almost constant between 15° and 45° and decrease significantly from 45° to 75° in the ascendant trajectory. Values increase at 90° because of a lack of cutting speed and decrease at 105°. In the descendant trajectory, values decrease slightly between 105° and 135° and remain almost constant between 105° and 165°. In conventional milling, similar results were obtained. As a general trend, lower roughness values were obtained for conventional milling than for climb milling in the ascendant trajectory, and higher roughness values were obtained for conventional milling than for climb milling in the descendant trajectory.

**Figure 5:**
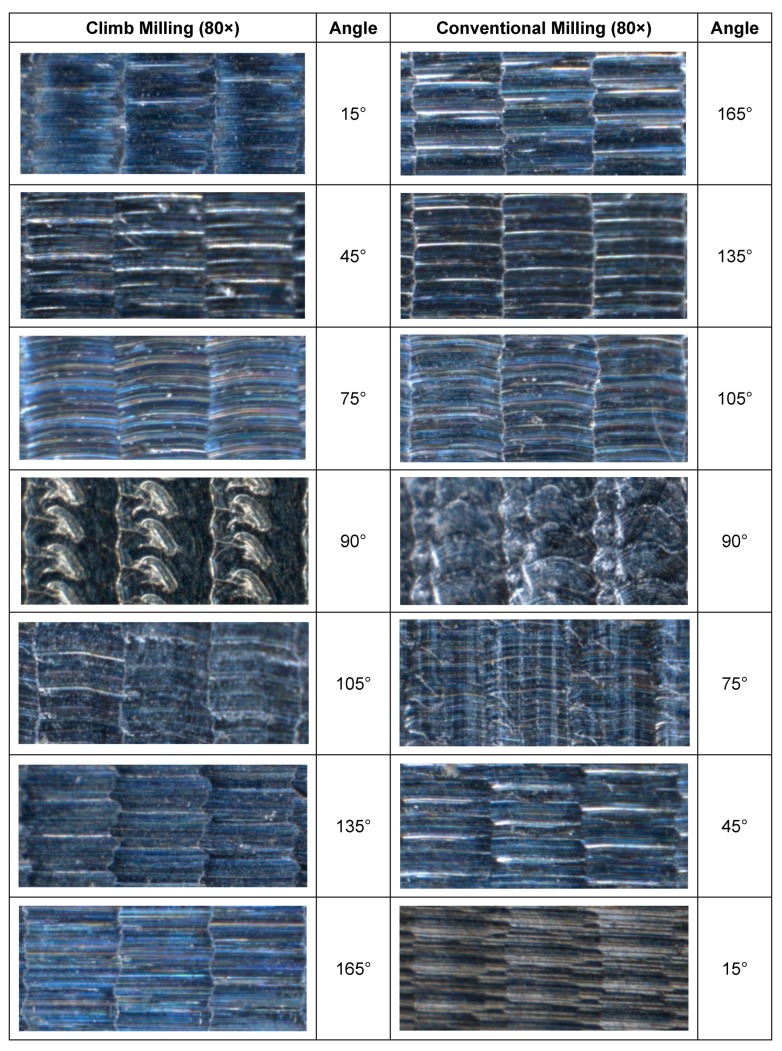
Machined surfaces corresponding to experiment 16.

In Figure 5, machined surfaces of experiment 16 are presented.

In experiment 16, for each angle considered, surface topography obtained in climb milling is similar to that obtained in conventional milling. However, Table 3 shows that in general, when *f_z_* trajectory is ascendant, roughness is lower for conventional milling (165° to 135°) than for climb milling (15° to 45°). On the other hand, when *f_z_* trajectory is descendant, roughness is lower for climb milling (135° to 165°) than for conventional milling (45° to 15°). At 90°, instead of straight cutting marks, semicircular cutting marks are observed, suggesting that the tool does not cut properly because of zero cutting speed [1,2]. 

## 4. Models for Surface Roughness

In this study, first the main cutting conditions that minimize *Ra* and *Rt* roughness parameters and their variability were selected. In addition to strategies and cutting conditions, inclination of the machined surface was considered, as there seems to be a lack of knowledge on the attained roughness in the manufacturing process of molds when different slopes have to be machined. Within the range of *a_e_* and *f_z_* values studied, surface topography is mainly determined by roughness in the transversal direction, which is perpendicular to tool marks in the feed *f_z_* direction. Along tool marks the roughness level is remarkably low, since *f_z_* < *a_e_* [24,25]. For this reason, 2D roughness was studied along the transversal direction (perpendicular to tool marks).

Vivancos et al. [15] previously analyzed this behavior by considering four factors (*a_p_*, *a_e_*, *f_z_* and *v_c_*) in regression models and by taking into account average roughness values in the whole workpiece without considering influence of each position angle separately. In order to obtain a more accurate analysis, it is necessary to consider the effect of each surface slope on obtained roughness, which is one of the core points of this work. Vakondios et al. [20] considered surface inclination in regression models for average maximum height of the profile, *Rz*. In the present study, regression analysis was carried out considering not only cutting conditions but also position angle of the surface on two different roughness parameters, *Ra* and *Rt*. Both regression and neural networks models were obtained. All regression analyses were carried out using Statgraphics® Centurion XVI. Regarding neural network models, the results found in this study were obtained by using the Neural Network Toolbox^TM^ (São Paulo, Brazil) of Matlab^TM^ (Mathworks, Natick, MA, USA). In addition, the optimal cutting strategy between climb and conventional milling was selected for different cutting conditions and inclination angles.

### 4.1. Regression Models and Analysis of Arithmetic Average Roughness, Ra

*Ra* was modeled by means of regression analysis, taking into account variability due to cylindrical geometry of the workpiece studied in this present work. In order to model the behavior of *Ra* for both manufacturing strategies (climb milling and conventional milling), second-order models were selected after analyzing *p*-values obtained from the lack-of-fit test performed with the first order modeling (3.0 × 10^−12^ and 3.04 × 10^−4^, respectively). Since these *p*-values for the lack-of-fit are less than 0.05, there is a statistically significant lack-of-fit at the 95.0% confidence level, which means that first order models do not adequately represent the data. R^2^ and adjusted-R^2^ were 68.43% and 67.25% for climb milling, respectively, while R^2^ and adjusted-R^2^ were 69.40% and 68.26% for conventional milling, respectively.

**Figure 6:**
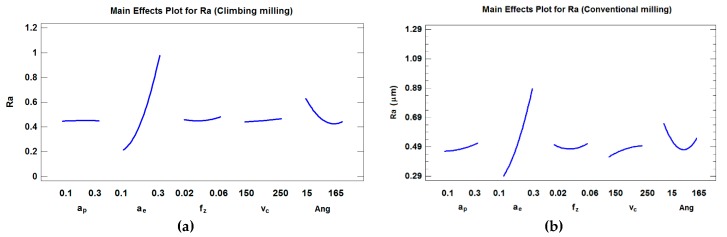
Main effects plot for *Ra* (considering the position angle) in (**a**) climb milling and (**b**) conventional milling.

Since there is lack of fit with the first order model, second order models were considered. For *Ra* in climb milling, the R^2^ and adjusted-R^2^ are 79.48% and 77.64%, respectively, and equations were obtained so that adjusted- R^2^ is maximized. Four main effects (*a_e_*, *Ang*, ***v_c_*** and *f_z_)* turned out to be relevant in the model in order to obtain the highest adjusted-R^2^. Parameters *a_e_* and *a_e_* ^2^ turned out to be the most significant for a confidence level of 95% (α = 0.05) (*p*-values ≤ 0.01). As can be observed in Figure 6, surface roughness remains almost constant with respect to *a_p_*, ***v_c_*** and *f_z_*. Moreover, it can be shown that *Ra* has a quadratic tendency with regard to *a_e_*, where *a_e_* is the parameter that most influences *Ra*. Therefore, minimization of *a_e_* will lead to a reduction in roughness values. This can be attributed to the fact that *a_e_* determines width of machining marks, and in addition *f_z_* values are low. In the study the rest of the factors are kept at their central values. Moreover, it can be shown that *Ra* has a quadratic tendency with regard to *Ang*.

Equations (1) and (2) show the proposed modeling for *Ra* using both climb and conventional milling. For *Ra* in conventional milling, R^2^ and adjusted-R^2^ are 76.52% and 73.84%, respectively. Four main effects (*a_e_*, v_c_, *Ang* and *a_p_*,) turned out to be relevant in the model in order to obtain the highest adjusted-R^2^. Similar to the results obtained in climb milling, *a_e_* and *a_e_*^2^ were the most significant factors at a confidence level of 95% (α = 0.05) (*p-*values ≤ 0.01).

As can be observed in Figure 6, surface roughness has a quadratic tendency with regard to *a_e_*, and a slight slope with respect to both *a_p_* and *v_c_*. In this case, factor *f_z_* was not significant in the model that provides the highest adjusted-R^2^. Moreover, a quadratic tendency with regard to the angle was observed. Conventional milling (Figure 6b) follows a similar tendency to climb milling (Figure 6a) regarding *a_e_*, which is the most significant parameter. However, this influence is smaller than that obtained in climb milling.

(1) $$\begin{array}{cc} Ra\_Climb= & 0.379019-1.42452\times \;a_{p}-0.661785\times a_{e}-0.194863\times f_{z}-0.00189356\times v_{c}\\ & \;+0.00239249\times Ang-0.269701\times a_{p}^{2}-1.87232\times a_{p}\times a_{e}+1.01875\times a_{p}\\ & \;\times f_{z}+0.00672571\times a_{p}\times v_{c}+0.00594601\times a_{p}\times Ang\;+14.563\times a_{e}^{2}\\ & \;-17.0402\times a_{e}\times f_{z}+0.00579143\times a_{e}\times v_{c}-0.01599\times a_{e}\times Ang+50.1135\\ & \;\times f_{z}^{2}+0.0134214\times f_{z}\times v_{c}-0.030112\times f_{z}\times Ang+0.00000135074\times v_{c}^{2}\\ & \;-0.0000158617\times v_{c}\times Ang\;+0.0000152557\times Ang^{2} \end{array}\;R^{2}=79.48\%\;\mathrm{Adj}-R^{2}=77.14\%$$

(2) $$\begin{array}{cc} Ra\_Convent= & 0.344666+0.376502\times a_{p}+0.060028\times a_{e}-9.41584\times f_{z}+0.00130472\times v_{c}\\ & \;-0.0054896\times Ang+1.19437\times a_{p}^{2}-2.68455\times a_{p}\times a_{e}-0.828125\times a_{p}\times f_{z}\\ & \;-0.000939821\times a_{p}\times v_{c}+0.00201332\times a_{p}\times Ang+9.38685\times a_{e}^{2}\\ & \;-22.5379\times a_{e}\times f_{z}+0.00357411\times a_{e}\times v_{c}+0.00083119\times a_{e}\times Ang\\ & \;+80.4796\times f_{z}^{2}+0.0369991\times f_{z}\times v_{c}+0.00481444\times f_{z}\times Ang\\ & \;-0.00000658429\times v_{c}^{2}+6.90684\times 10^{-7}\times v_{c}\times Ang+0.0000217804\times Ang^{2} \end{array}\;R^{2}=76.53\%\;\mathrm{Adj}-R^{2}=73.84\%$$

### 4.2. Regression Models and Analysis of Maximum Peak-to-Valley Roughness Rt

Similar to the results obtained for *Ra*, the behavior of *Rt* was modeled, taking into account variability due to cylindrical geometry of the workpiece studied. In order to model the behavior of *Rt* in both manufacturing strategies (climb and conventional milling), second-order models were selected after analyzing *p*-values obtained from the lack-of-fit test performed with the first order modeling (5.52 × 10^−5^ and 1.14 × 10^−26^, respectively). In all cases, obtained equations were simplified in order to obtain models with the highest adjusted-R^2^.

**Figure 7:**
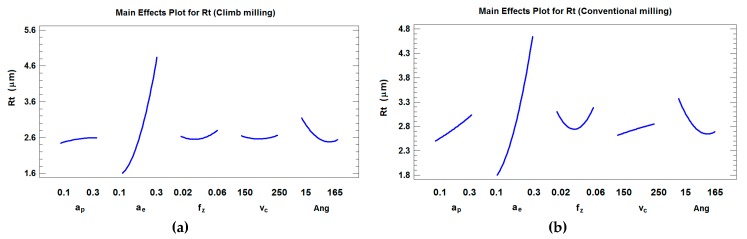
Main effects plot for *Rt* (considering the position angle) in (**a**) climb milling and (**b**) conventional milling.

For *Rt*, in climb milling the R^2^ and adjusted-R^2^ are 78.04% and 75.53%, respectively. Four main effects (*a_e_*, *Ang*, *f_z_* and *a_p_*,) were present in the model in order to obtain the highest adjusted-R^2^. The parameters *a_e_* and *a*_e_^2^ were the most important parameters at a confidence level of 95% (α = 0.05) *(p-*values ≤ 0.01) (Figure 7a). For *Rt*, in conventional milling R^2^ and adjusted-R^2^ were 63.03% and 58.80%, respectively. Three main effects (*a_e_*, *a_p_*, and *Ang*) were present in the model in order to obtain the highest adjusted-R^2^. Similar to the result obtained for climb milling, *a_e_* and *a_e_*^2^ were the most important parameters at a confidence level of 95% (α = 0.05) *(p-*values ≤ 0.01) (Figure 7b). Equations (3) and (4) show the regression analysis for *Rt*, taking the angle into account and considering both climb milling and conventional milling.

(3) $$\begin{array}{cc} Rt\_Climb= & 1.812-1.27897\times a_{p}-1.59259\times a_{e}+11.4864\times f_{z}-0.0153509\times v_{c}\\ & \;+0.0158467\times Ang-4.28786\times a_{p}^{2}-2.19768\times a_{p}\times a_{e}+23.8839\times a_{p}\times f_{z}\\ & \;+0.00801286\times a_{p}\times v_{c}+0.0177314\times a_{p}\times Ang+64.6309\times a_{e}^{2}-114.621\\ & \;\times a_{e}\times f_{z}+0.0180725\times a_{e}\times v_{c}-0.0715339\times a_{e}\times Ang+390.288\times f_{z}^{2}\\ & \;-0.0134036\times f_{z}\times v_{c}-0.197239\times f_{z}\times Ang+0.0000386847\times v_{c}^{2}\\ & \;-0.0000518305\times v_{c}\times Ang+0.0000505297\times Ang^{2} \end{array}\;R^{2}=78.04\%\;\mathrm{Adj}-R^{2}=75.53\%$$

(4) $$\begin{array}{cc} Rt\_Convent= & 4.04698+4.45254\times a_{p}+0.0118769\times a_{e}-100.633\times f_{z}-0.00524991\times v_{c}\\ & \;-0.021916\times Ang+2.41174\times a_{p}^{2}-7.9592\times a_{p}\times a_{e}-38.4004\times a_{p}\times f_{z}\\ & \;-0.00523589\times a_{p}\times +0.0158633\times a_{p}\times Ang+46.2882\times a_{e}^{2}-155.4\times a_{e}\times f_{z}\\ & \;+0.00680554\times a_{e}\times v_{c}+0.0256059\times a_{e}\times Ang+1002.59\times f_{z}^{2}+0.263647\\ & \;\times f_{z}\times v_{c}+0.0953535\times f_{z}\times Ang-0.00000371912\times v_{c}^{2}-0.0000202955\times v_{c}\\ & \;\times Ang+0.0000517796\times Ang^{2} \end{array}\;R^{2}=63.02\%\;\mathrm{Adj}-R^{2}=58.8\%$$

As can be observed in Figure 6, *a_e_* is the most influential parameter on *Rt* in both climb and conventional milling, which is similar to the results obtained for *Ra* in the present paper and for *Rz* parameter in other works [19,20]. Surface roughness has a quadratic behavior with respect to a*_e_* in climb and conventional milling, and a slight slope with both *a_p_* and *v_c_* in conventional milling. In climb milling, surface roughness remains almost constant with respect to *a_p_*, *f_z_* and ***v_c_***. The fact that a_e_ has a greater influence on roughness than *f_z_* in ball-end milling processes can be explained by the fact that at low radial depth of cut *a_e_*, the influence of feed per tooth *f_z_* is minimized by the tool performing very close successive passes in the *a_e_* direction. Very close parallel grooves will be obtained. Thus, very similar roughness values will be achieved regardless of *f_z_* employed for the same *a_e_* value [24].

### 4.3. ANN Modeling for Ra and Rt

An artificial neural network (ANN) was also employed in this present study for modeling both *Ra* and *Rt*. This ANN was made up of an input layer, a hidden layer, and an output layer. The neural network considered in this work has a 5-1-4 configuration, which corresponds with five inputs (the four cutting conditions uses in regression analysis (*a_e_*, *a_p_*, *f_z_*, and *v_c_*) and the position angle of the surface (*Ang*), which is related to the slope of the surface to be machined. The network has one neuron in the hidden layer, and four outputs, one for each of the roughness parameters and machining strategies considered. Equation (5) shows the roughness parameters *Ra* and *Rt* for both machining strategies as a function of *a_p_*, *a_e_, f_z_*, ***v_c_***, and *Ang*.

(5) $$\left[\begin{array}{c} {\mathrm{Ra}}_{\mathrm{Climb}}.\\ {\mathrm{Ra}}_{\mathrm{Conv}}.\\ {\mathrm{Rt}}_{\mathrm{Climb}}.\\ {\mathrm{Rt}}_{\mathrm{Conv}}. \end{array}\right]=\frac{1}{1+e^{-\left(-0.0449{\times a}_{p}-1.90361{\times a}_{e}+0.0855{\times f}_{z}-0.111{\times v}_{c}+0.266\;\times \;Ang+1.899\right)}}\left[\begin{array}{c} -2.3137\\ -2.6528\\ -1.9889\\ -1.9144 \end{array}\right]+\left[\begin{array}{c} 1.3164\\ 1.8037\\ 1.0583\\ 1.1065 \end{array}\right]$$

where Climb. corresponds to climb milling and Conv. Corresponds to conventional milling.

The design of experiments, previously shown in Table 2, was used to train the ANN. It was decided to choose one neural network with four outputs, since the results obtained were similar to those obtained for independent networks for each output. With this ANN a correlation value of 0.914 was obtained. This value is similar to that obtained by other authors with ANN models [19]. Hence, ANN 5-1-4 provides a relatively simple model with high precision, which in a compact way allows approximation of *Ra* and *Rt* roughness parameters in both machining strategies studied. This might be attributed to the fact that roughness parameters are related and they show similar variability. 

### 4.4. Optimal Manufacturing Strategy Selection

**Figure 8:**
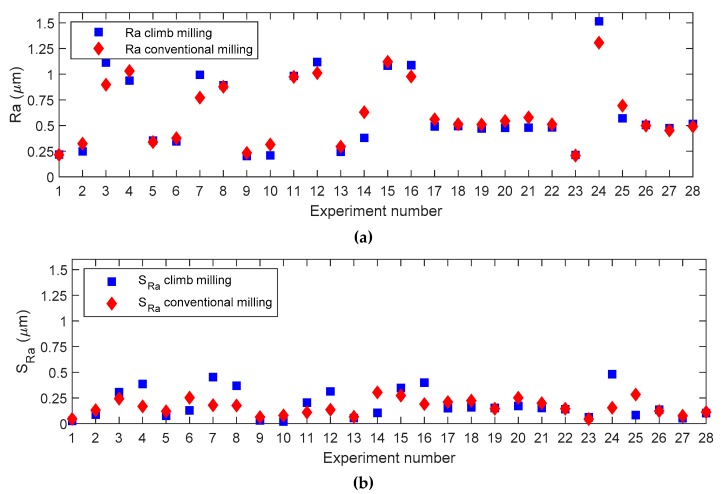
Experimental deviation plots for *Ra* considering both manufacturing strategies: (**a**) Mean, (**b**) standard deviation.

In order to compare both machining strategies, a diagram of both average roughness values and standard deviations of roughness values obtained at different inclination angles for the 28 experiments considered is shown in Figure 7 and Figure 8, for *Ra* and *Rt*, respectively. From these figures it is possible to determine which machining strategy is more appropriate for the cutting conditions selected in this present work.

Figure 8a shows that average *Ra* values are very similar for both climb milling and conventional milling, if the same experiment is taken into consideration. However, in surfaces with variable inclinations, such as those found in injection molds, it is interesting not only to minimize roughness average values, but also its variability for different inclination angles. This will lead to a more uniform surface roughness. Then, in order to minimize variability (Figure 8b), the use of conventional milling is recommended in experiments 3, 4, 7, 8, 11, 12, 15, 16, and 24. Those experiments have a general tendency to exhibit high *a_e_* values (*a_e_* = 0.3 mm). Using climb milling is recommended in experiments 2, 5, 6, 9, 10, 14, 17, 18, 20, 21, 25, and 27, which in general correspond with low and medium *a_e_* values (*a_e_* = 0.1 mm and *a_e_* = 0.2 mm, respectively).

**Figure 9:**
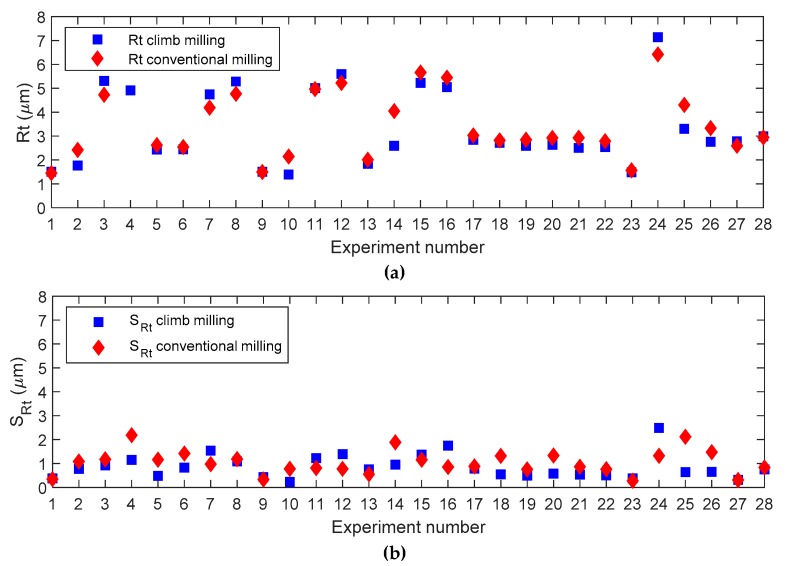
Experimental deviation plots for *Rt* considering both manufacturing strategies: (**a**) Mean, (**b**) standard deviation.

For the remainder of experiments, similar values were obtained for both conventional and climb milling. Figure 9a also shows that average *Rt* values are similar for both machining strategies. However, variability (Figure 9b) determines that conventional milling is recommended in experiments 7, 11, 12, 13, 15, 16, and 24. As a general trend, those experiments correspond to high *a_e_* values (a*_e_* = 0.3 mm), with high ***v_c_*** values (***v_c_*** = 250 m/min). Climb milling is recommended in experiments 2, 3, 4, 5, 6, 10, 14, 17, 18, 19, 20, 21, 22, 25, and 26, which correspond to maximum *a_e_* with minimum ***v_c_***, minimum *ae* with maximum ***v_c_***, or medium *a_e_* with medium ***v_c_*** values. For the rest of experiments, the values obtained are similar.

*Ra* and *Rt* average values do not vary significantly between climb and conventional milling. Given that mold manufacturers require roughness uniformity at different inclination angles of the machined surface, the most appropriate process will be chosen between conventional and climb milling, taking variability into account in the experiments studied (Table 4). Therefore, a manufacturing strategy will be selected that minimizes variability of roughness values in different angular positions. If *Ra* and *Rt* show opposite tendencies, a manufacturing strategy will be preferred that minimizes *Rt*, since *Ra* is a high-averaging parameter and, therefore, tends to mask errors on the machined surface. This does not happen with *Rt*. In the case where both strategies lead to the same *Rt* variability, then the strategy minimizing *Ra* variability will be chosen.

Table 4 summarizes the type of machining strategy that is recommended for each cutting condition and for each cutting strategy. The table shows that in 17 of 28 cutting conditions tested, climb milling is preferred. Conventional milling is only preferred in 8 cutting conditions, which in general corresponds with high *a_e_* with high ***v_c_***. For the rest of the experiments, it makes no difference whether one or the other machining strategy is used. As was stated earlier, minimization of *Rt* has priority with respect to minimization of *Ra*. Std means standard deviation of roughness values for the different inclination angles. Conv. Milling stands for conventional milling.

Regarding influence of angle, for both strategies (climb and conventional milling), when angle increases roughness decreases. However, it should be taken into account that high angles in climb milling (descendant trajectory) correspond to low angles in conventional milling (descendant trajectory), and low angles in climb milling (ascendant trajectory) correspond to high angles in conventional milling (ascendant trajectory). With all this, it is recommended to use climb milling in descendant trajectories and conventional milling in ascendant trajectories.

## 5. Conclusions

In the present study, as a general tendency climb milling is preferred to conventional milling. In general, conventional milling is only recommended at a high radial depth of cut with high cutting speed values. In order to reduce roughness values, in ascendant trajectories conventional milling is preferred and in descendant trajectories climb milling is recommended.

From the results obtained, it was determined that radial depth of cut was the most relevant factor on *Ra* and *Rt* for both climb and conventional milling. Axial depth of cut, cutting speed and feed per tooth have a slight influence on roughness within the range studied in this study. Regression models for average roughness showed high adjusted-R^2^ values (above 73%) in all cases. Moreover, a correlation value of 0.914 was obtained with the neural network model employed.

Experimental roughness values obtained with both strategies (climb and conventional milling) were similar. However, in complex surfaces with variable inclination, such as those of injection molds, it is recommended not only to minimize roughness average values, but also its variability for different inclination angles. This will lead to more uniform surfaces. In the present study, it was found that the standard deviation of roughness parameters varies depending on the machining strategy chosen, for the different experiments carried out. 

## Figures and Tables

**Table 1:** Tool details.

Tool Type	End Mill VC2SBR0300 KOBELCO Series MIRACLE (Kobe Steel, Chūō-ku, Kobe, Japan)
Tool material	(Al, Ti)N coated micro grain carbide
Number of flutes	2
Diameter (mm)	6

**Table 2:** Low and high levels for factors *a_e_*, *a_p_*, *f_z_* and *v_c_*.

Levels	*a_p_*	*a_e_*	*f_z_*	*v_c_*
Low	0.100	0.100	0.020	150.0
High	0.300	0.300	0.060	250.0

**Table 3:** *Ra* and *Rt* values using climb milling (Figure 3a) and conventional milling (Figure 3b) for experiment 16 in different angular positions.

Parameter	Climb Milling (Figure 3a)							Conventional Milling (Figure 3b)						
	15°	45°	75°	90°	105°	135°	165°	15°	45°	75°	90°	105°	135°	165°
***Ra* (μm)**	1.56	1.68	0.88	1.23	0.80	0.73	0.75	1.22	1.06	0.70	1.05	0.83	0.83	1.15
***Rt* (μm)**	7.04	7.05	3.82	6.54	4.17	3.08	3.65	5.41	4.72	4.52	7.05	6.00	5.18	5.25

**Table 4:** Optimal machining strategy selection.

*a_p_* (mm)	*a_e_* (mm)	*f_z_* (mm)	*v_c_* (m/min)	Minimum (Std *Ra*)	Minimum (Std *Rt*)
0.1	0.1	0.02	150	Conv./Climb	Conv./Climb
0.3	0.1	0.02	150	Climb Milling	Climb Milling
0.1	0.3	0.02	150	Conv. Milling	Climb Milling
0.3	0.3	0.02	150	Conv. Milling	Climb Milling
0.1	0.1	0.06	150	Climb Milling	Climb Milling
0.3	0.1	0.06	150	Climb Milling	Climb Milling
0.1	0.3	0.06	150	Conv. Milling	Conv. Milling
0.3	0.3	0.06	150	Conv. Milling	Conv. / Climb
0.1	0.1	0.02	250	Climb Milling	Conv./Climb
0.3	0.1	0.02	250	Climb Milling	Climb Milling
0.1	0.3	0.02	250	Conv. Milling	Conv. Milling
0.3	0.3	0.02	250	Conv. Milling	Conv. Milling
0.1	0.1	0.06	250	Conv./Climb	Conv. Milling
0.3	0.1	0.06	250	Climb Milling	Climb Milling
0.1	0.3	0.06	250	Conv. Milling	Conv. Milling
0.3	0.3	0.06	250	Conv. Milling	Conv. Milling
0.2	0.2	0.04	200	Climb Milling	Conv./Climb
0.2	0.2	0.04	200	Climb Milling	Climb Milling
0.2	0.2	0.04	200	Conv./Climb	Climb Milling
0.2	0.2	0.04	200	Climb Milling	Climb Milling
0.039	0.2	0.04	200	Climb Milling	Climb Milling
0.361	0.2	0.04	200	Conv./Climb	Climb Milling
0.2	0.039	0.04	200	Conv./Climb	Conv./Climb
0.2	0.361	0.04	200	Conv. Milling	Conv. Milling
0.2	0.2	0.008	200	Climb Milling	Climb Milling
0.2	0.2	0.072	200	Conv./Climb	Climb Milling
0.2	0.2	0.04	119.641	Climb Milling	Conv./Climb
0.2	0.2	0.04	280.359	Conv./Climb	Conv./Climb
